# Mass cytometry of early immune remodeling after mRNA booster vaccination reveals exploratory CD4 naive T-cell associations with documented breakthrough infection

**DOI:** 10.3389/fcimb.2026.1966930

**Published:** 2026-10-15

**Authors:** Xuanjing Yu, Xinyu Jiang, Yupeng Xue, Yuqi Ji, Chunmei Li, Zijie Scott Zhang, Rui Cheng

**Affiliations:** 1 Bio-X Center for Interdisciplinary Innovation, Yunnan University, Kunming, Yunnan, China; 2 Yunnan Vaccine Laboratory, Kunming, Yunnan, China; 3 Southwest United Graduate School, Kunming, Yunnan, China

**Keywords:** breakthrough infection, early immune response, H3K27ac, m6A (N6-methyladenosine), mass cytometry, mRNA vaccine, SARS-CoV-2, systems immunology

## Abstract

**Background:**

Messenger RNA (mRNA) vaccines rapidly engage innate and adaptive immunity, but the systemic immune changes occurring within 24 h of vaccination remain incompletely understood. We investigated whether early cellular and global H3K27ac/m6A changes were associated with subsequent documented breakthrough infection (BTI).

**Methods:**

We performed an exploratory longitudinal systems-immunology analysis of an outcome-balanced subset of 124 participants from a randomized trial of COVID-19 mRNA booster vaccination (62 BTI and 62 non-BTI). Peripheral blood mononuclear cells collected at Day 0 and Day 1 were profiled by high-dimensional mass cytometry. Seventeen major immune-cell populations were assessed for changes in relative abundance, marker profiles, and global H3K27ac/m6A signals. Neutralizing-antibody responses were measured through Day 180.

**Results:**

Within 24 h, booster vaccination induced rapid lineage-specific remodeling of circulating immune composition. Classical and intermediate monocytes showed positive shifts in CLR-transformed relative abundance, whereas pDCs, NK cells, and selected CD8 and unconventional T-cell populations showed negative shifts. CD4 T-cell populations remained comparatively stable on this relative-composition scale. Early H3K27ac and m6A changes correlated with abundance changes in selected innate and T-cell populations. Associations with later humoral immunity were limited: after exclusion of antibody measurements obtained on or after documented BTI, no association between early H3K27ac/m6A changes and Day 14 or Day 180 neutralizing-antibody responses remained significant after multiple-testing correction. Major-cell abundance trajectories did not differ significantly by subsequent BTI status. In contrast, BTI was associated with smaller Day 0-to-Day 1 changes in CD4_Tn IFN–Th1 chemokine, cytotoxic–Th1-associated, and CCL2–CCR2-associated marker scores and in the m6A readout.

**Conclusions:**

mRNA booster vaccination induces rapid, cell-type-specific immune remodeling within 24 h. Major-cell abundance changes were broadly shared across BTI groups, whereas early CD4_Tn marker-associated and m6A signal changes were associated with subsequent documented BTI. These findings support further evaluation of dynamic early immune states as correlates of heterogeneity in vaccine responses.

## 1 Introduction

Messenger RNA (mRNA) vaccines have emerged as an effective platform for inducing protective immunity against severe acute respiratory syndrome coronavirus 2 (SARS-CoV-2). Lipid nanoparticle (LNP)-encapsulated, nucleoside-modified mRNA enables transient antigen expression while simultaneously engaging innate immune pathways that facilitate the development of adaptive immunity (Pardi et al., 2018; Teijaro and Farber, 2021; Alameh et al., 2021). Large-scale clinical trials demonstrated that the BNT162b2 and mRNA-1273 vaccines provided high levels of protection against symptomatic COVID-19, accompanied by robust neutralizing-antibody and antigen-specific T-cell responses (Polack et al., 2020; Baden et al., 2021; Sahin et al., 2020). Subsequent studies further demonstrated that mRNA vaccination induces coordinated CD4 and CD8 T-cell responses, persistent germinal-center reactions, T follicular helper-cell responses, and durable B- and T-cell memory (Painter et al., 2021; Oberhardt et al., 2021; Turner et al., 2021; Mudd et al., 2022; Goel et al., 2021). These observations underscore that the immunogenicity of mRNA vaccines is not determined by a single arm of immunity, but rather emerges from coordinated interactions between early innate sensing and subsequent adaptive immune activation. Nevertheless, protection against symptomatic infection varies substantially among vaccinated individuals, and breakthrough infections (BTIs) remain common, particularly as circulating antibody levels decline and viral antigenic evolution reduces neutralization sensitivity (Levin et al., 2021; Bergwerk et al., 2021; Khoury et al., 2021; Gilbert et al., 2022; Cromer et al., 2022). Although the downstream humoral and cellular responses to mRNA vaccination have been extensively characterized, how the human peripheral immune system changes during the first 24 h after vaccination, particularly across multiple innate and adaptive immune-cell compartments, remains incompletely understood.

The first 24–72 h after vaccination represent a particularly dynamic phase during which antigen delivery and innate immune recognition are translated into systemic immune activation. Studies across multiple vaccine platforms have established that molecular and cellular signatures arising within the first few days after immunization can be associated with the magnitude and quality of subsequent adaptive responses (Querec et al., 2009; Pulendran et al., 2010; Nakaya et al., 2011; Pulendran, 2014; Rechtien et al., 2017). In the context of SARS-CoV-2 mRNA vaccination, systems-level studies have revealed rapid induction of interferon-associated transcriptional programs, inflammatory monocyte responses, cytokine and chemokine production, and dynamic changes in circulating innate-cell populations (Arunachalam et al., 2021; Bergamaschi et al., 2021; Li et al., 2022; Takano et al., 2022). In particular, early changes involving monocytes, dendritic cells, NK cells, interferon-responsive programs, and IFN-γ-inducible chemokines have been linked to vaccine immunogenicity or subsequent antibody responses (Arunachalam et al., 2021; Bergamaschi et al., 2021; Takano et al., 2022). Experimental studies further indicate that LNP- and mRNA-derived signals initiate distinct but interacting inflammatory and type I interferon programs at the injection site, with IFN-β signaling contributing to the recruitment and activation of dendritic cells and subsequent cellular immunity (Kim et al., 2024; Alameh et al., 2021). Booster vaccination can also rapidly mobilize effector T-cell populations while preserving the underlying memory pool (Oberhardt et al., 2021; Reinscheid et al., 2022). Collectively, these observations suggest that the earliest post-vaccination period is not simply a transient inflammatory phase, but a biologically informative transition during which local innate recognition is propagated into systemic cellular responses. Day 1 therefore represents a biologically informative window for capturing the transition from immediate vaccine sensing to coordinated systemic immune activation, before the substantial expansion of downstream adaptive responses becomes dominant. However, a comprehensive cell-type-resolved characterization of this 24-h transition in the human peripheral immune system remains limited.

Rapid immune activation requires coordinated regulation at multiple molecular levels, including changes in chromatin activity and post-transcriptional RNA regulation. Acetylation of histone H3 at lysine 27 (H3K27ac) is enriched at active promoters and enhancers and is widely used as an indicator of active regulatory chromatin (Creyghton et al., 2010). During innate immune stimulation, macrophages undergo extensive remodeling of their enhancer landscape, including stimulus-dependent acquisition of active enhancer marks, enabling rapid and cell-state-specific transcriptional responses (Kaikkonen et al., 2013; Ostuni et al., 2013). More persistent epigenetic remodeling can also modify the responsiveness of innate immune cells to subsequent stimulation, forming part of the molecular basis of trained or innate immune memory (Saeed et al., 2014; Netea et al., 2016, 2020; Lercher et al., 2024). In parallel with chromatin regulation, N6-methyladenosine (m6A), the most prevalent internal modification of eukaryotic mRNA, provides a dynamic layer of epitranscriptomic regulation by influencing RNA stability, processing, localization, and translation (Shulman and Stern-Ginossar, 2020). m6A-dependent regulation has been implicated in innate antiviral immunity, including control of type I interferon production and interferon-stimulated gene expression (Winkler et al., 2019; McFadden et al., 2021). Thus, H3K27ac and m6A represent complementary regulatory layers through which immune cells may rapidly modify transcriptional and post-transcriptional states in response to vaccination. Despite these advances, it remains largely unknown whether mRNA vaccination alters global H3K27ac and m6A signals across distinct circulating immune-cell populations during the immediate response, how such signal changes relate to simultaneous shifts in cellular phenotype and abundance, and whether inter-individual variation in these early trajectories is associated with subsequent documented BTI status.

To address these questions, we performed a longitudinal systems-immunology analysis of 124 participants from a previously reported randomized clinical trial of COVID-19 mRNA booster vaccination, using a frequency-balanced subset comprising 62 participants with documented breakthrough infection and 62 with no documented COVID-19 during follow-up. High-dimensional mass cytometry was used to profile peripheral blood mononuclear cells collected immediately before vaccination (Day 0) and 24 h after vaccination (Day 1), providing a cell-type-resolved view of the 24-h circulating immune response. In parallel, neutralizing-antibody responses against a panel of SARS-CoV-2 variants were longitudinally measured from Day 0 to Day 180. We first characterized Day 0-to-Day 1 changes in the abundance and molecular phenotype of major innate and adaptive immune-cell populations, and then examined cell-type-specific marker-associated modules together with the chromatin-associated H3K27ac signal and the global m6A signal. We further evaluated whether early H3K27ac- and m6A-associated changes correlated with relative-abundance changes and subsequent neutralizing-antibody responses. Finally, by comparing the early immune trajectories of participants grouped by subsequent documented BTI status, we sought to determine whether variation in the immediate post-vaccination response was associated with subsequent documented BTI status. This integrated framework allowed us to address three related questions: how the circulating immune system is reorganized within 24 h of mRNA vaccination, how global H3K27ac and m6A signal changes correlate with early cellular phenotypic and compositional changes, and whether variation in these early response trajectories differs by subsequent documented BTI status.

## 2 Materials and methods

### 2.1 Study participants and clinical trial design

This study was performed as an exploratory immunological analysis using a subset of participants from a previously reported randomized, active-controlled, observer-blinded clinical trial (ClinicalTrials.gov identifier: NCT05907044) (Yu et al., 2024). The original trial evaluated the immunogenicity and safety of the XBB.1.5/Alpha-Beta bivalent RQ3027, BA.2/BA.5-Alpha/Beta bivalent RQ3025, and Alpha/Beta monovalent RQ3013 mRNA boosters in healthy adults previously vaccinated against COVID-19. Detailed information regarding the study design, eligibility criteria, vaccination procedures, and clinical follow-up has been described previously.

COVID-19 cases were prospectively recorded from Day 7 through Day 180 after booster vaccination in the parent trial and were defined according to World Health Organization COVID-19 case definitions. COVID-19 cases were ascertained according to the trial surveillance procedures, which included participant-performed SARS-CoV-2 rapid antigen testing for COVID-19-like illness and PCR confirmation when available. For the present analysis, participants with a documented COVID-19 case during this prespecified follow-up period were classified as having breakthrough infection (BTI), whereas participants without a documented COVID-19 case during the same period were classified as non-BTI. Because surveillance was illness-triggered, non-BTI denotes the absence of documented COVID-19 rather than confirmed absence of all SARS-CoV-2 infection.

A total of 124 participants were included in the present study, comprising 62 BTI and 62 non-BTI participants. Peripheral blood samples collected at multiple time points after vaccination were analyzed by mass-cytometric immune profiling and pseudovirus neutralization assays. The original clinical trial was approved by the Scientific Review Committee and Ethics Committee of the Affiliated Hospital of Yunnan University (ID: 2023113), and all participants provided written informed consent. The study was conducted in accordance with the Declaration of Helsinki, International Council for Harmonization Good Clinical Practice guidelines, and local regulatory requirements.

### 2.2 Pseudovirus neutralization assay

Serum neutralizing antibody responses against SARS-CoV-2 variants were evaluated using a vesicular stomatitis virus (VSV)-based pseudovirus neutralization assay. Briefly, serum samples were heat-inactivated at 56 °C for 30 min and serially diluted before incubation with standardized VSV-SARS-CoV-2 pseudoviruses. Pseudoviruses representing the prototype strain and multiple SARS-CoV-2 variants, including Alpha, Beta, and Omicron subvariants (BA.4/5, BF.7, BQ.1.1, XBB.1.5, XBB.1.16, XBB.1.9.1 and JN.1), were used in the neutralization assay. Assay availability varied by visit; in particular, JN.1 neutralization was measured at Days 0, 14, 28, 90, and 180 but was not available at Day 7. Serum-pseudovirus mixtures were incubated with HEK293T-ACE2 cells, and infection levels were quantified by measuring Renilla luciferase activity. The 50% neutralizing antibody titers (NC50) were estimated using nonlinear regression as previously described (Nie et al., 2020).

For the longitudinal neutralizing-antibody comparison shown in **Figure 1B**, participants who experienced BTI before a given sampling time point were excluded from the analysis at that time point and all subsequent time points. Accordingly, only antibody measurements obtained before documented BTI contributed to the longitudinal BTI-group summaries and between-group comparisons. A corresponding time-dependent censoring rule was also applied to the **Figure 4** correlation analysis, as described in Section 2.11.

**Figure 1:**
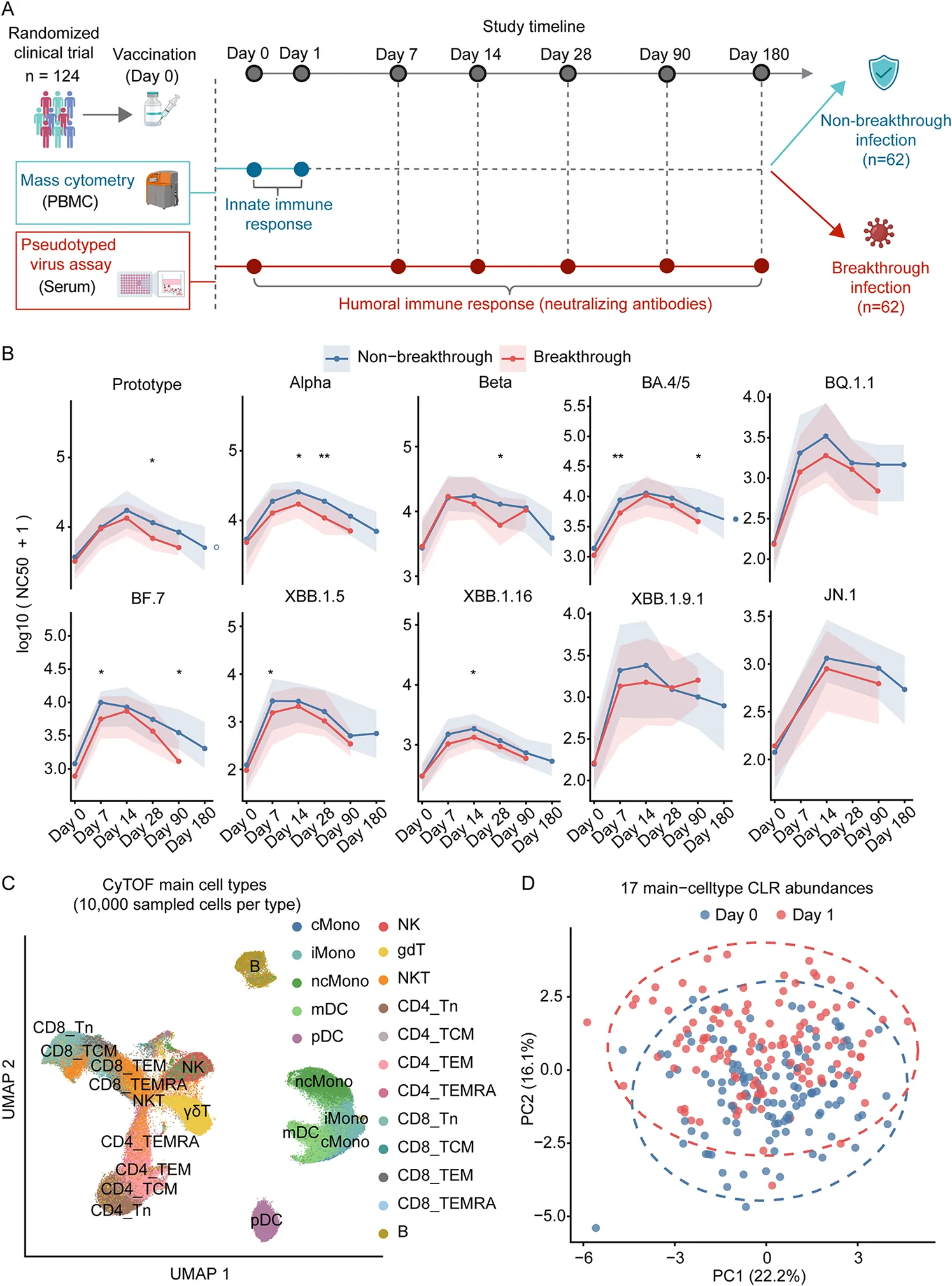
Study design and longitudinal humoral and cellular immune profiling following booster vaccination. **(A)** Schematic overview of the study design. A total of 124 participants received booster vaccination at Day 0 and were selected for the outcome-balanced subset according to documented BTI status (non-BTI, n = 62; BTI, n = 62) during follow-up. Peripheral blood mononuclear cells (PBMCs) collected at Days 0 and 1 were analyzed by mass cytometry to characterize the early cellular immune response. Serum samples collected at Days 0, 7, 14, 28, 90, and 180 were analyzed using pseudotyped-virus neutralization assays. **(B)** Longitudinal serum neutralizing-antibody responses against the prototype strain and the Alpha, Beta, BA.4/5, BQ.1.1, BF.7, XBB.1.5, XBB.1.16, XBB.1.9.1, and JN.1 variants in non-BTI and BTI participants. Neutralizing activity is presented as log10(NC50 + 1). Points and lines indicate the median, and shaded bands represent the interquartile range (IQR; Q1–Q3) at each time point. For this longitudinal analysis, antibody measurements obtained on or after documented BTI were excluded at that visit and all subsequent visits. Accordingly, the numbers of participants with at least one available neutralizing-antibody measurement at each visit were 62/62 (non-BTI/BTI) at Day 0, 62/62 at Day 7, 62/54 at Day 14, 62/31 at Day 28, 62/8 at Day 90, and 62/0 at Day 180. Exact sample sizes varied slightly across individual strains because of assay availability and occasional missing measurements; complete time-point-, group-, and strain-level sample-size information is provided in **Supplementary Table S8**. Between-group comparisons at each time point were performed using two-sided Wilcoxon rank-sum tests followed by Benjamini–Hochberg false-discovery-rate correction. **(C)** UMAP visualization of the 17 manually gated major immune-cell populations. For descriptive visualization, up to 10,000 cells were randomly sampled from each major population, pooled, and projected into UMAP space after dimensionality reduction and correction for experimental date. Cells are colored according to their FlowJo-based manually gated identities; no additional clustering was performed in this pooled analysis. The corresponding manual gating strategy is shown in **Supplementary Figure S1**. **(D)** Principal-component analysis based on CLR-transformed abundances of all 17 major immune-cell populations at Day 0 and Day 1. No feature selection based on longitudinal change was performed, and the CLR abundance matrix contained no missing values; therefore, no imputation was applied. Each point represents one participant sample, and dashed ellipses summarize the distributions at the two visits. PC1 and PC2 explained 22.2% and 16.1% of the total variance, respectively. Asterisks indicate BH-adjusted significance: *FDR< 0.05, **FDR< 0.01. BTI, breakthrough infection; non-BTI, no documented breakthrough infection; PBMC, peripheral blood mononuclear cell; UMAP, uniform manifold approximation and projection; CLR, centered log-ratio; PCA, principal-component analysis.

### 2.3 Mass cytometry sample preparation

Peripheral blood mononuclear cells (PBMCs) were isolated from peripheral blood samples collected during the clinical trial and cryopreserved in liquid nitrogen until further analysis. For mass cytometry experiments, cryopreserved PBMCs were rapidly thawed and washed in Cell Staining Buffer (CSB; calcium- and magnesium-free PBS [E607009, Sangon Biotech, Shanghai, China] supplemented with 2% fetal bovine serum [FBS, Thermo Fisher, C11875500BT]) before downstream processing. After thawing, cells were allowed to recover for 1 h at 37 °C in a humidified 5% CO_2_ incubator. No ex vivo antigenic or mitogenic stimulation was applied, because the aim was to characterize the unstimulated post-vaccination intracellular state. Cells were then incubated for an additional 2 h at 37 °C and 5% CO₂ with brefeldin A (BFA; Sigma-Aldrich, B5936-200UL) at a final concentration of 20 μg/mL and monensin (Protein Transport Inhibitor; Waters, 554724) at 0.67 μL/mL before subsequent mass-cytometry staining. To reduce nonspecific antibody binding, cells were incubated with Fc receptor blocking reagent (BioLegend, 422302) prior to antibody staining. Cell viability was assessed using a 195Pt-labeled cisplatin-based viability reagent (P03536-0000, Hangzhou PuKang Medical Technology Co., Ltd.).

### 2.4 Mass cytometry sample staining

For sample multiplexing, PBMC samples were individually labeled with an in-house CD45-based mass cytometry barcoding system using six metal isotope-conjugated anti-CD45 antibodies (106Cd, 110Cd, 111Cd, 113Cd, 114Cd, and 116Cd). A combinatorial 6-choose-3 barcoding strategy was applied, generating 20 unique barcode combinations that enabled simultaneous processing of multiple samples in a single mass cytometry experiment.

Following barcoding, pooled samples were stained with a metal-conjugated surface antibody cocktail (**Supplementary Table S1**) targeting immune lineage and differentiation markers. Cells were subsequently fixed and permeabilized using the Transcription Factor Staining Buffer Set (Invitrogen, Cat. No. 00-5523), followed by staining with a metal-conjugated intracellular antibody cocktail (**Supplementary Table S2**) targeting signaling molecules, cytokines, H3K27ac, and m6A. After intracellular staining, cells were labeled with an iridium-based DNA intercalator (P03534-0000, Hangzhou PuKang Medical Technology Co., Ltd.) for DNA-content measurement and prepared for mass cytometry acquisition.

CyTOF acquisition was performed prospectively across 39 experimental dates/barcode pools in the broader paired-sample dataset. Each pool contained up to 20 samples. Paired Day 0 and Day 1 samples from the same participant were processed within the same experimental date and barcode pool. Sample-level CyTOF experimental-date/barcode-pool assignments for the samples included in the present analysis are provided in **Supplementary Table S9**.

All staining and barcoding antibodies were individually titrated during panel development. Each titration series included an unstained blank control consisting of cells from the same preparation that underwent the same experimental processing, including fixation and permeabilization where applicable, but received no antibody staining. These titration and blank-control data are provided in **Supplementary Figure S2** and were used to assess signal-to-background characteristics and select the final working antibody concentrations.

Because CyTOF detects metal isotopes rather than overlapping fluorescence-emission spectra, no fluorescence-style post-acquisition compensation matrix was applied. Potential mass-cytometry-specific channel interference, including isotopic impurity and M + 16 oxide formation, was considered prospectively during antibody-to-metal channel assignment.

### 2.5 Mass cytometry acquisition and data preprocessing

Mass cytometry data were acquired using the MSFLO mass cytometry system (Hangzhou PuKang Medical Technology Co., Ltd.). Raw FCS files were first normalized using the bead normalization module (Finck et al., 2013) to correct for short-term signal fluctuations and long-term intensity drift during data acquisition. This bead-based normalization addressed technical acquisition drift and did not normalize the H3K27ac signal to total histone/chromatin content or the m6A signal to total cellular RNA content. Accordingly, H3K27ac and m6A were analyzed as global antibody-derived signals rather than normalized modification fractions. Pooled samples were subsequently deconvoluted into individual sample files using the Single Cell Debarcoding tool (Zunder et al., 2015) based on barcode signal patterns.

Following debarcoding, individual FCS files were imported into FlowJo (version 10.10.0) for quality control and manual gating. Single cells were identified based on DNA content and event length, followed by exclusion of debris, doublets, and dead cells. Seventeen major immune-cell populations were defined using established lineage and differentiation markers: classical monocytes (cMono), intermediate monocytes (iMono), non-classical monocytes (ncMono), myeloid dendritic cells (mDCs), plasmacytoid dendritic cells (pDCs), natural killer (NK) cells, γδ T cells, NKT-like cells, CD4 naive T cells (CD4_Tn), CD4 central memory T cells (CD4_TCM), CD4 effector memory T cells (CD4_TEM), CD4 terminally differentiated effector memory T cells (CD4_TEMRA), CD8 naive T cells (CD8_Tn), CD8 central memory T cells (CD8_TCM), CD8 effector memory T cells (CD8_TEM), CD8 terminally differentiated effector memory T cells (CD8_TEMRA), and B cells. Functional/cytokine/chemokine markers were not used to define those immune-cell populations. These manually gated populations formed the basis for all subsequent abundance, phenotypic, and computational analysis. Because CD1d tetramers and invariant-NKT-specific TCR markers were not included in the CyTOF panel, the CD3⁺CD56⁺ population was designated as NKT-like cells rather than invariant NKT (iNKT) cells. Within the CD4 T-cell compartment, CD4_Tn cells were operationally defined as CD3⁺CD4⁺CD27⁺CD45RA⁺ cells. Because CCR7 and CD45RO were not included in the CyTOF panel, this population is referred to throughout the revised manuscript as a phenotypically defined CD4_Tn compartment rather than as a functionally or transcriptomically purified conventional CD4 naive T-cell population.

### 2.6 CyTOF analysis subset

For the CyTOF analysis, BTI and non-BTI participants were frequency-balanced at a 1:1 ratio within strata defined by sex, vaccine product, and age category to achieve balanced group composition. The resulting outcome-balanced CyTOF subset comprised 62 BTI and 62 non-BTI participants. All 124 participants had paired Day 0 and Day 1 samples passing the quality-control procedures used for the present analysis, yielding 248 CyTOF samples in total. BTI status was defined at the participant level according to documented COVID-19 occurrence during the Day 7-to-Day 180 follow-up period described above and remained fixed for all analysis. Day 0 and Day 1 samples were used for baseline and early post-vaccination CyTOF analysis, respectively. At the sample level, each participant sample was treated as one biological observation; individual cells were not treated as independent replicates.

Participant selection for the present outcome-balanced analysis was performed after CyTOF acquisition; thus, the 248 analyzed samples were distributed across the pre-existing experimental batches rather than constituting separately assembled barcode pools. Potential association between experimental date and subsequent BTI status in the 124-participant subset was evaluated using a permutation test of the experimental-date × BTI contingency table; no systematic association was detected (permutation P = 0.89).

### 2.7 CyTOF phenotypic preprocessing and fine-cell subclustering

The 17 major immune-cell populations were defined by manual gating in FlowJo, as described above. For downstream phenotypic analysis, barcode, DNA-intercalator, and viability channels were excluded, and marker intensities were transformed using an arcsinh transformation with a cofactor of 5.

Fine-scale cell subpopulations were identified separately within each manually gated major cell population using Scanpy (Wolf et al., 2018). For each population, transformed phenotypic-marker expression was subjected to principal component analysis, followed by Harmony-based correction for experimental date (Korsunsky et al., 2019). A nearest-neighbor graph was constructed from the batch-corrected low-dimensional representation, and unsupervised Leiden community detection was applied to identify phenotypically distinct subclusters (Traag et al., 2019). UMAP was used to visualize the resulting fine-cell landscape. The resulting clusters were treated as fine-scale cell subpopulations for downstream abundance and marker-expression analysis. To avoid single-cell pseudoreplication, marker expression for each sample within each major cell type or fine subpopulation was summarized as the median transformed signal.

Harmony correction was restricted to low-dimensional representations used for visualization and subclustering and was not applied to the sample-level CLR abundances, median marker intensities, or composite marker scores used for statistical inference.

### 2.8 Descriptive dimensionality-reduction analysis

For visualization of the major-cell phenotypic landscape, up to 10,000 cells were randomly sampled from each manually gated major cell population and pooled. Transformed marker intensities were standardized, followed by principal component analysis, Harmony-based correction for experimental date, construction of a nearest-neighbor graph, and UMAP embedding. Cells were labeled according to their manually gated major cell population; no additional clustering was performed in this pooled analysis.

For sample-level visualization, CLR-transformed abundance profiles across all 17 major immune-cell populations were used for principal-component analysis. No feature selection based on Day 0-to-Day 1 change was performed. The 248 × 17 CLR abundance matrix contained no missing values; therefore, no imputation was required. CLR features were standardized before PCA, and samples were colored by visit for descriptive visualization.

### 2.9 Cell abundance and marker-expression analysis

For major cell populations and within-lineage subpopulations, sample-level cell abundances were transformed using the centered log-ratio (CLR) transformation to account for the compositional nature of abundance data. For sample i and cell population j, CLR abundance was calculated as


$${\text{CLR}}_{\text{ij}}=\ln\left({\text{n}}_{\text{ij}}+0.5\right)-\frac{1}{\text{K}}\sum_{\text{k}=1}^{\text{K}}\ln\left({\text{n}}_{\text{ik}}+0.5\right)$$

where n_ij_ denotes the cell count for population j in sample i, and K denotes the number of cell populations included in the corresponding compositional vector. Natural logarithms were used. A pseudocount of 0.5 was included to accommodate potential zero cell counts. In the 248 × 17 major-population count matrix analyzed here, however, no zero counts were present (minimum count, 13 events). Across samples, the total number of events assigned to the 17 major immune-cell populations had a median of 201,069 (IQR, 150,632–256,638; range, 43,959–457,422). No additional minimum-event threshold was imposed on the 17 major populations in the primary analysis. Because all sample–population combinations contained observed cells, sample-level median marker intensities were defined for all major-population combinations. Per-sample event counts and quality-control summaries are provided in **Supplementary Table S7**.

Marker expression was summarized as the sample-level median arcsinh-transformed intensity of each marker. Group differences were assessed at Day 0 and Day 1, with the effect direction consistently defined as breakthrough infection (BTI) minus non-BTI. Longitudinal responses were quantified for each participant as the Day 1-minus-Day 0 change (Δ). For cell abundance, this was defined as $\Delta \text{CLR}={\text{CLR}}_{\text{Day}1}-{\text{CLR}}_{\text{Day}0}$, and Δ values were compared between BTI and non-BTI groups.

Between-group comparisons at Day 0, Day 1, and for Δ were performed using two-sided Wilcoxon rank-sum tests. Within-group Day 0-to-Day 1 changes were evaluated using two-sided paired Wilcoxon signed-rank tests. Effect sizes were expressed on the analyzed scale as median between-group differences or median within-participant changes in CLR abundance or transformed marker intensity, as appropriate. For abundance visualizations, the displayed longitudinal effect was the median participant-level ΔCLR, ensuring that visualization and statistical inference were reported on the same transformed scale. Raw P values and Benjamini–Hochberg (BH)-adjusted false discovery rate (FDR) values were retained in the result tables, and displayed statistical annotations were based on BH-FDR values. BH correction was applied within prespecified hypothesis families defined for each analysis, with the exact family size reported in the corresponding Methods section, figure legend, and supplementary result table.

### 2.10 Marker-associated module scoring

Marker-associated module scores were calculated from sample-level median marker expression within each major cell type and fine cell population. For each marker within a cell type, pooled Day 0 and Day 1 values across participants were robustly standardized by subtracting the median and dividing by the median absolute deviation; when the median absolute deviation was zero, the interquartile-range- or standard-deviation-based estimate was used. Multi-marker module scores were the equal-weight mean of the available standardized constituent markers. The modules comprised type I interferon/JAK-STAT-associated score (IFN-α and pSTAT1), IFN/Th1 chemokine-associated score (CXCL9 and CXCL10), pro-inflammatory cytokine-associated score (TNF-α, IL-1β, and IL-6), CCL2/CCR2-associated score (MCP-1 and CCR2), antigen presentation/co-stimulation (HLA-DR and CD86), activation/proliferation (CD69, CD38, and Ki-67), and cytotoxic/Th1-associated score (Granzyme B, IFN-γ, and CD57). CXCR3, pS6, arginase-1, IL-4, m6A, and H3K27ac were retained as individual readouts rather than averaged into composite modules. Group and longitudinal comparisons of marker-module scores used the same Wilcoxon testing and BH-FDR framework described above. For the primary screen of BTI-associated early feature changes shown in **Figure 5B**, participant-level Day 1-minus-Day 0 changes (Δ) were compared between BTI and non-BTI participants for all 13 prespecified feature readouts across all 17 major immune-cell populations. The BH correction family therefore comprised 221 tests (13 features × 17 cell populations). Nominal P values and BH-adjusted q values were calculated across this complete 221-test family. **Figure 5D** did not constitute a separate inferential analysis or multiple-testing family; it was used to provide a focused visualization of the four CD4_Tn features among the five feature–cell-population combinations that met the BH-FDR< 0.05 threshold in the **Figure 5B** screen. The P and q values displayed in **Figure 5D** were carried forward directly from the corresponding **Figure 5B** tests without re-testing or re-adjustment. Module labels describe their marker composition and should not be interpreted as direct assays of cellular function.

### 2.11 Association of early H3K27ac and m6A signal changes with subsequent neutralizing-antibody responses

Spearman rank-correlation analysis was used to evaluate associations between early changes in H3K27ac or m6A and subsequent neutralizing-antibody responses. For each of the 17 major immune-cell populations, early marker change was quantified at the participant level as the Day 1-minus-Day 0 change in median transformed H3K27ac or m6A signal. Neutralizing-antibody responses were expressed as the log2 fold change from Day 0 to Day 14 or from Day 0 to Day 180 for 10 viral strains: the prototype strain, Alpha, Beta, BA.4/5, BQ.1.1, BF.7, XBB.1.5, XBB.1.16, XBB.1.9.1, and JN.1. To minimize confounding by breakthrough infection, antibody measurements obtained on or after documented BTI were excluded at the corresponding time point and all subsequent time points; thus, only pre-BTI antibody measurements contributed to these analyses. Spearman correlations were calculated separately for each cell population–marker–strain combination using pairwise-complete observations, resulting in 340 tests per antibody-response interval (17 cell populations × 2 global modification-associated readouts × 10 strains). Nominal two-sided P values were retained, and BH correction was applied separately across the complete 340 correlations within each time-point analysis.

### 2.12 Software and reporting

Downstream CyTOF phenotypic preprocessing and subclustering were performed in Python using Scanpy and Harmony. Sample-level statistical analysis, visualization, and marker-module scoring were performed in R. All tests were two-sided. Unless otherwise stated, statistical significance was defined as BH-FDR< 0.05. Exact sample sizes, effect estimates, nominal P values, and BH-FDR values are provided in the corresponding supplementary tables where applicable.

## 3 Results

### 3.1 Study cohort and longitudinal immune profiling after booster vaccination

This exploratory immunological study included 124 participants from a previously reported randomized, active-controlled, observer-blinded clinical trial (ClinicalTrials.gov identifier: NCT05907044). The outcome-balanced subset comprised 62 participants with documented breakthrough infection (BTI) and 62 with no documented COVID-19 (non-BTI) during follow-up (**Figure 1A**). Baseline demographic characteristics, including age, sex distribution, and body mass index, were descriptively similar between the two groups (**Table 1**). In addition, pre-booster neutralizing antibody titers against the prototype strain and tested SARS-CoV-2 variants were similar between BTI and non-BTI participants (**Table 1**), indicating similar baseline serum neutralization profiles for the tested strains.

**Table 1:** Clinical characteristics of the outcome-balanced CyTOF subset.

Characteristics	Breakthrough infection	Non-breakthrough infection
N	62	62
Age, years		
Mean, SD	30.0 ± 6.7	29.8 ± 6.6
Median, IQR	28.0 (24.0, 35.0)	29.0 (24.0, 35.0)
Sex		
Female	28 (45.2%)	28 (45.2%)
Male	34 (54.8%)	34 (54.8%)
Body-mass index, n (mean BMI, kg/m²)		
<18.5: underweight	12 (17.6)	4 (17.5)
18.5-24.9: healthy weight	34 (22.1)	43 (22.1)
25.0-29.9: overweight	10 (27.6)	14 (27.1)
≥30.0: obesity	6 (32.3)	1 (31.6)
Neutralizing antibodies against SARS-CoV-2 variants pre-boosters, GMT (95% CI)		
Prototype	3424.5 (2558.1-4584.2)	3395.9 (2628.1-4387.9)
Alpha	4412.3 (3202.1-6079.8)	4715.1 (3663.2-6069.2)
Beta	2629.4 (1834.4-3768.9)	2762.6 (2082.8-3664.2)
BA.4/5	985.0 (693.2-1399.5)	1209.5 (933.1-1567.7)
BF.7	884.5 (627.3-1247.1)	958.1 (742.9-1235.6)
BQ.1.1	145.7 (116.5-182.1)	163.1 (126.8-209.7)
XBB.1.5	89.7 (65.9-122.0)	103.9 (78.3-137.7)
XBB.1.16	265.9 (211.6-334.0)	301.0 (232.6-389.7)
XBB.1.9.1	136.3 (100.7-184.5)	168.3 (124.1-228.1)
JN.1	127.9 (97.2-168.2)	107.0 (82.1-139.5)

SD, standard deviation; IQR, interquartile range; GMT, geometric mean titer; 95% CI, 95% confidence interval.

Percentages for categorical variables were calculated within each study group. BMI categories were defined using standard adult cutoffs.

Following booster vaccination, longitudinal serum neutralization was assessed using pseudovirus neutralization assays at Days 0, 7, 14, 28, 90, and 180, and high-dimensional mass cytometry profiling was performed using PBMC samples collected at Days 0 and 1 (**Figure 1A**).

Neutralizing-antibody titers rose rapidly after vaccination against all tested SARS-CoV-2 variants in both groups (**Figure 1B**). The response trajectories overlapped substantially between BTI and non-BTI individuals, although several variant-specific differences were observed. Significant differences after Benjamini–Hochberg correction were detected for Alpha at Days 14 and 28, BA.4/5 at Days 7 and 90, BF.7 at Days 7 and 90, Beta and prototype strain at Day 28, XBB.1.16 at Day 14, and XBB.1.5 at Day 7 (BH-FDR< 0.05). No significant differences were observed for BQ.1.1, JN.1, or XBB.1.9.1 at the analyzed time points (BH-FDR ≥ 0.05). Thus, between-group differences were confined to selected strain–time-point combinations rather than being consistent across the panel.

FlowJo-based manual gating identified 17 major circulating immune-cell populations, including monocyte and dendritic-cell subsets, NK and NKT-like cells, conventional and unconventional T-cell populations, and B cells (**Supplementary Figure S1**). To visualize the global phenotypic relationships among these manually defined populations, cells were subsampled from each population and projected into a UMAP space after dimensionality reduction and batch correction (**Figure 1C**). The UMAP visualization showed the phenotypic organization of the manually defined major immune-cell populations. Principal-component analysis based on CLR-transformed abundances of all 17 major immune-cell populations showed partially shifted but substantially overlapping Day 0 and Day 1 distributions (**Figure 1D**). PC1 and PC2 explained 22.2% and 16.1% of the total variance, respectively. No feature selection based on longitudinal change was performed, and the CLR abundance matrix contained no missing values; therefore, no imputation was applied. Together, these findings indicate that booster vaccination induced rapid but heterogeneous reorganization of the circulating immune landscape, prompting further investigation of the cellular compartments and marker-level changes underlying this early response.

### 3.2 Booster vaccination induces innate-cell redistribution and lineage-specific marker changes

We first examined six major innate-cell populations that showed clear early compositional changes (**Figure 2A**). All six populations showed significant Day 0-to-Day 1 changes in CLR abundance after multiple-testing correction. Classical and intermediate monocytes showed the largest positive shifts in CLR-transformed relative abundance, whereas pDCs and NK cells showed prominent negative shifts. More modest changes were observed for mDCs and non-classical monocytes. These results describe an early circulating innate-cell profile characterized by increased representation of classical and intermediate monocytes accompanied by reduced representation of circulating pDC and NK compartments.

**Figure 2:**
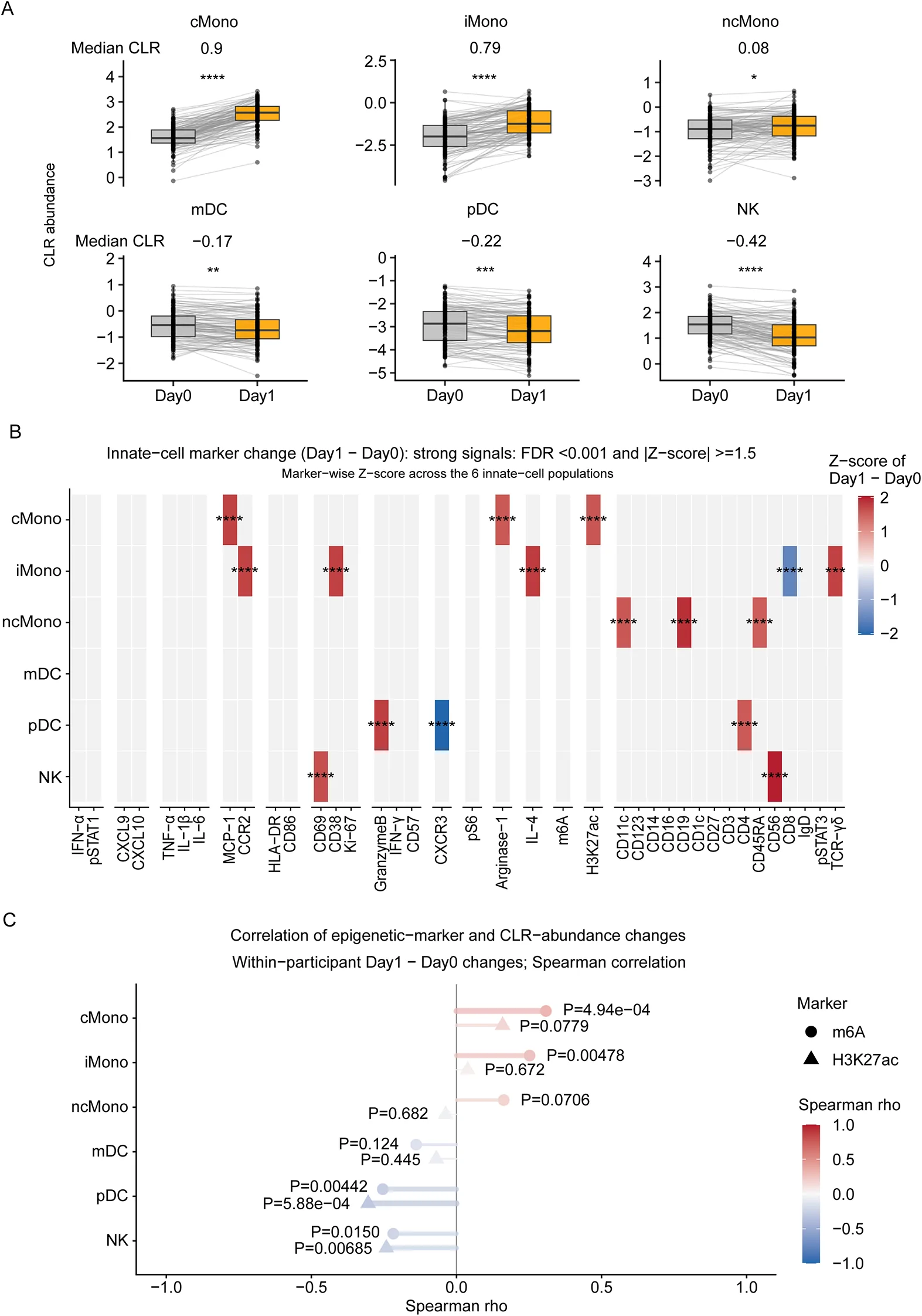
Rapid innate-cell redistribution is accompanied by lineage-specific marker-level and global H3K27ac/m6A signal changes. **(A)** Paired changes in CLR-transformed abundance from Day 0 to Day 1 across six major innate-cell populations. Lines connect paired samples from the same participant, and boxplots summarize the distributions at each time point. Values above the plots indicate the median within-participant change in CLR-transformed abundance (ΔCLR = CLR_Day1_ − CLR_Day0_). Day 0-to-Day 1 differences were assessed using two-sided paired Wilcoxon signed-rank tests, with BH-FDR correction across the six cell populations. **(B)** Heatmap of marker-level changes from Day 0 to Day 1 across the six innate-cell populations. For each marker, within-participant changes were standardized across cell populations and are shown as marker-wise Z-scores. Red and blue indicate relative increases and decreases, respectively. Colored tiles identify strong marker–cell population signals meeting both BH-FDR< 0.001 and |Z-score| ≥ 1.5. **(C)** Spearman correlations between within-participant Day 1-minus-Day 0 changes in the global modification-associated readouts m6A or H3K27ac and the corresponding changes in CLR-transformed cell abundance. Circles represent m6A and triangles represent H3K27ac. Color indicates the Spearman correlation coefficient, and labels indicate raw two-sided P values. BH-FDR correction was performed across the 12 cell population–marker correlations. Asterisks in panels A and B indicate adjusted significance: *FDR< 0.05, **FDR< 0.01, ***FDR< 0.001, and ****FDR< 0.0001. cMono, classical monocytes; iMono, intermediate monocytes; ncMono, non-classical monocytes; mDC, myeloid dendritic cells; pDC, plasmacytoid dendritic cells; NK, natural killer cells; CLR, centered log-ratio; m6A, N6-methyladenosine; H3K27ac, histone H3 lysine 27 acetylation.

The abundance changes were accompanied by distinct, lineage-restricted alterations in intracellular and phenotypic marker signals (**Figure 2B**). Classical monocytes showed prominent changes in MCP-1, arginase-1, and H3K27ac, whereas intermediate monocytes displayed changes involving CCR2, CD38, IL-4, and several lineage-associated markers. Non-classical monocytes showed prominent changes in CD11c and CD45RA, whereas pDCs showed changes involving granzyme B and CXCR3; NK cells showed marked changes in CD69 and CD56. The complete marker-level heatmap also contained isolated lineage-incongruent signals, including CD19 in non-classical monocytes and CD4 in pDCs. Because these markers were not used to define cell identity and their biological specificity cannot be established from the present data, we retained them in the complete visualization for transparency but did not interpret them as lineage-specific functional changes.

To determine whether the global modification-associated readouts were linked to changes in cell abundance, we correlated within-participant Day 1-minus-Day 0 changes in m6A or H3K27ac with the corresponding CLR-abundance changes (**Figure 2C**). The labels in the figure show raw Spearman P values, while BH-FDR values were calculated across the 12 cell-population-marker correlations and retained in the statistical output. m6A changes correlated positively with cMono (P = 4.94 × 10^-4^) and iMono abundance (P = 0.00478), but inversely with pDC (P = 0.00442) and NK abundance (P = 0.0150). H3K27ac changes were also inversely correlated with pDC (P = 5.88 × 10^-4^) and NK abundance (P = 0.00685). All six associations remained significant after BH correction across the 12 tests (q ≤ 0.030), whereas no significant correlations were observed for ncMono or mDC populations. These associations indicate that early H3K27ac and m6A-associated changes correlated with relative-abundance changes in selected innate-cell populations, with opposing relationships in monocytes versus pDC and NK cells.

### 3.3 Early T-cell remodeling is concentrated in CD8 and unconventional T-cell compartments

We next extended the analysis to 10 conventional and unconventional T-cell populations (**Figure 3A**). In contrast to the pronounced innate-cell changes, the four major CD4 T-cell populations—CD4_Tn, CD4_TCM, CD4_TEM, and CD4_TEMRA—showed comparatively stable abundance between Days 0 and 1. Negative shifts in CLR-transformed relative abundance were concentrated in selected CD8 and unconventional T-cell populations, whereas the four major CD4 T-cell populations remained comparatively stable on this relative-composition scale. These findings suggest that early changes in T-cell composition were more evident in CD8 and unconventional T-cell populations rather than the bulk CD4 compartment.

**Figure 3:**
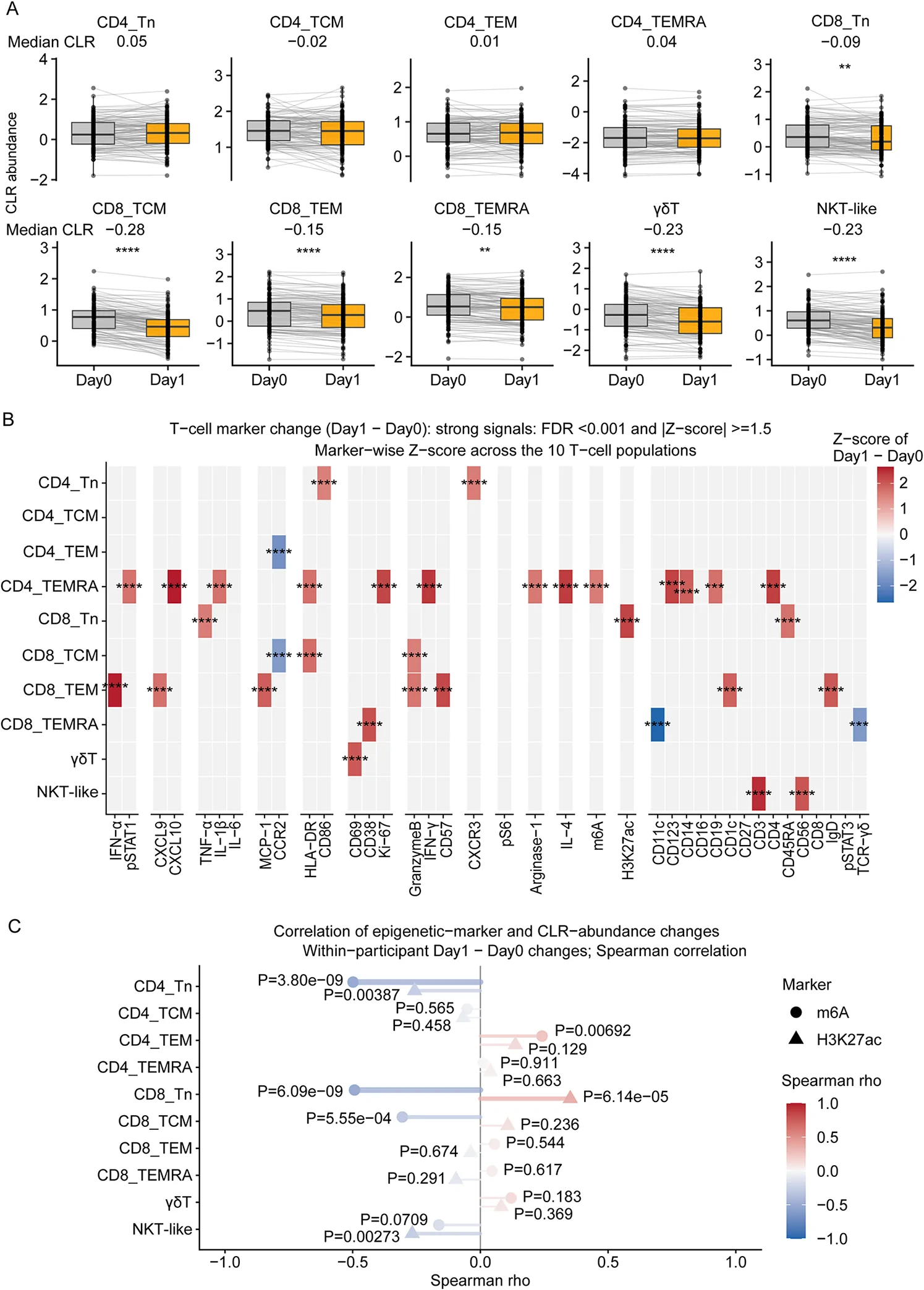
Early T-cell remodeling shows differentiation-state-specific changes in abundance, molecular phenotype, and global H3K27ac/m6A signals. **(A)** Paired changes in CLR-transformed abundance from Day 0 to Day 1 across 10 major T-cell populations. Lines connect paired samples from the same participant, and boxplots summarize the distributions at each time point. Values above the plots indicate the median within-participant change in CLR-transformed abundance (ΔCLR = CLR_Day1_ − CLR_Day0_). Day 0-to-Day 1 differences were assessed using two-sided paired Wilcoxon signed-rank tests, with BH-FDR correction across the ten cell populations. **(B)** Heatmap of marker-level Day 1-minus-Day 0 changes across the 10 T-cell populations. Changes were standardized marker-wise across populations and are presented as Z-scores. Red and blue indicate relative increases and decreases, respectively. Colored tiles identify strong marker–cell population signals meeting both BH-FDR< 0.001 and |Z-score| ≥ 1.5. Among the comparatively abundance-stable CD4_Tn population, CD86 and CXCR3 showed the most prominent changes. **(C)** Spearman correlations between within-participant changes in m6A or H3K27ac and changes in the corresponding T-cell CLR abundance. Circles represent m6A and triangles represent H3K27ac. Color indicates the Spearman correlation coefficient, and labels indicate raw two-sided P values. BH-FDR correction was performed across the 20 T-cell population–marker correlations. Asterisks in panels A and B indicate adjusted significance: *FDR< 0.05, **FDR< 0.01, ***FDR< 0.001, and ****FDR< 0.0001. Tn, naive T cells; TCM, central memory T cells; TEM, effector memory T cells; TEMRA, terminally differentiated effector memory T cells re-expressing CD45RA; γδT, γδ T cells; NKT-like, natural killer-like T cells; CLR, centered log-ratio; m6A, N6-methyladenosine; H3K27ac, histone H3 lysine 27 acetylation.

Despite their relatively stable abundance, several CD4 T-cell populations showed marker-level changes (**Figure 3B**). In CD4_Tn cells, CD86 and CXCR3 showed among the largest statistical changes; however, examination of the pre-standardization signal distributions indicated relatively low absolute signal intensities for both markers within this compartment (**Supplementary Figure S3**; **Supplementary Table S6**). These findings were therefore interpreted cautiously and were not considered direct evidence of altered co-stimulatory or migratory function. CD4_TEM cells displayed a prominent CCR2-associated alteration, whereas CD4_TEMRA cells exhibited broader marker-profile changes involving interferon-related signaling, inflammatory mediators, activation-associated markers, effector molecules, m6A, and differentiation-associated markers. Distinct combinations of inflammatory, cytotoxicity-associated, H3K27ac/m6A-associated, and differentiation-marker changes were also observed across CD8 T-cell subsets. Collectively, these findings indicate that relatively stable cell abundance can coexist with changes in measured marker profiles.

Correlations between H3K27ac/m6A signal changes and T-cell abundance further revealed differentiation-state-specific relationships (**Figure 3C**). Changes in both m6A and H3K27ac were inversely related to CD4_Tn abundance changes (P = 3.80 × 10^-9^ and P = 0.00387, respectively). m6A changes were positively associated with CD4_TEM abundance (P = 0.00692) but inversely associated with CD8_Tn (P = 6.09 × 10^-9^) and CD8_TCM abundance (P = 5.55 × 10^-4^). Conversely, H3K27ac changes were positively associated with CD8_Tn abundance (P = 6.14 × 10^-5^) and inversely associated with NKT-like abundance (P = 0.00273). After correction across the tested T-cell–marker combinations, the retained associations remained distributed across multiple differentiation states rather than converging on a single T-cell lineage. Collectively, these results demonstrate that early relative-abundance and global modification-signal changes were only partially correlated, and m6A and H3K27ac signal changes tracked distinct T-cell abundance trajectories.

### 3.4 Early H3K27ac and m6A signal changes show no associations with subsequent neutralizing-antibody responses after multiple-testing correction

Having identified cell-type-specific Day 0-to-Day 1 changes in global H3K27ac and m6A signals, we next investigated whether these early signal changes were associated with subsequent neutralizing-antibody responses. To avoid contamination of the antibody outcome by intervening infection, measurements obtained on or after documented BTI were excluded at each antibody time point.

At Day 14, 9 of the 340 cell population–marker–strain combinations showed nominal associations (raw P< 0.05) (**Figure 4A**), but none remained significant after BH-FDR correction across the complete 340-test family (**Supplementary Table S3**). The available sample size ranged from 113 to 116 participants across individual strain-specific comparisons.

**Figure 4:**
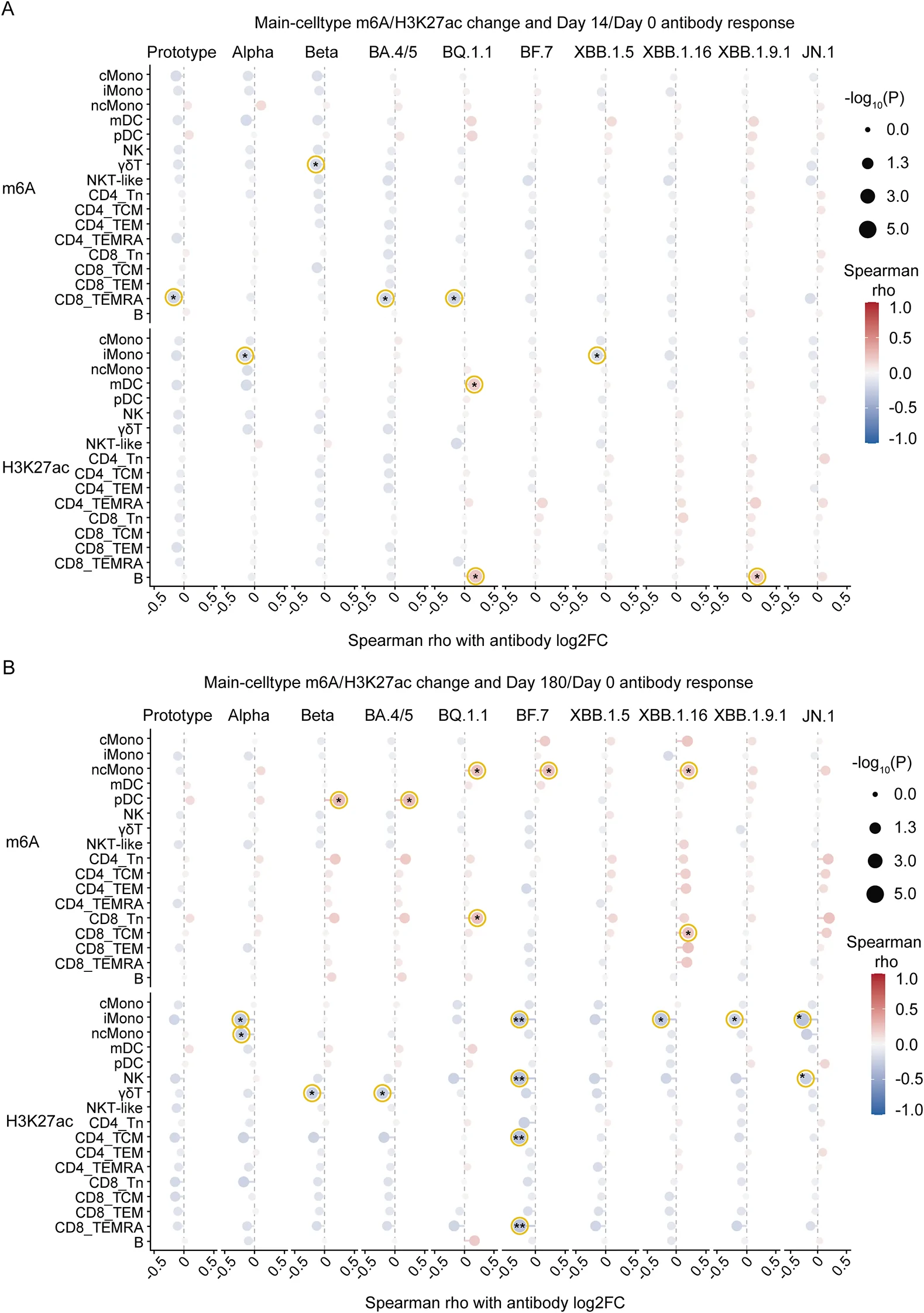
Early H3K27ac and m6A signal changes show limited associations with subsequent neutralizing-antibody responses. **(A, B)** Spearman correlations between Day 1-minus-Day 0 changes in m6A or H3K27ac across 17 major immune-cell populations and neutralizing-antibody responses against 10 SARS-CoV-2 strains, expressed as the log2 fold change from Day 0 to Day 14 **(A)** or from Day 0 to Day 180 **(B)**. Each point represents one cell population–marker–strain combination. Point color indicates the Spearman correlation coefficient, with red and blue representing positive and negative associations, respectively. Point size is proportional to −log10(P). Gold outlines and asterisks indicate nominal exploratory associations with raw two-sided P< 0.05 and do not denote significance after multiple-testing correction. Asterisks indicate *P< 0.05 and **P< 0.01. BH-adjusted q values were calculated across all 340 cell population–marker–strain correlations within each time-point analysis and are provided in **Supplementary Tables S3**, **S4**. No association remained significant at BH-FDR< 0.05 at either Day 14 or Day 180. m6A, N6-methyladenosine; H3K27ac, histone H3 lysine 27 acetylation; log2FC, log2 fold change; TCM, central memory T cells.

At Day 180, 21 nominal associations were observed (**Figure 4B**), but none remained significant after correction across the 340 tested correlations (**Supplementary Table S4**). The strongest nominal association was the inverse relationship between early H3K27ac-associated signal change in CD4_TCM cells and the BF.7 response (Spearman ρ = −0.339, raw P = 0.00696), but this association did not survive BH correction (q = 0.622). The available sample size ranged from 58 to 62 participants across comparisons.

Thus, after restricting the analysis to antibody measurements obtained before documented BTI and accounting for multiple testing, no early H3K27ac- or m6A-associated signal change showed statistically robust evidence of association with subsequent neutralizing-antibody responses at either Day 14 or Day 180.

### 3.5 Differential early CD4_Tn marker-associated and m6A signal changes are associated with subsequent BTI

Because early H3K27ac and m6A signal changes showed only limited relationships with subsequent neutralizing-antibody magnitude, we next asked whether the early cellular response differed by subsequent BTI status.

At the major-cell-population level, BTI and non-BTI participants exhibited broadly similar Day 0-to-Day 1 abundance responses (**Figure 5A**). Both groups showed prominent changes in monocyte, dendritic-cell, NK/NKT-like, CD8 T-cell, and B-cell compartments. However, no population showed a significant BTI versus non-BTI difference at Day 0 or Day 1, and no between-group difference in early abundance change remained significant after multiple-testing correction. Thus, the redistribution of major circulating immune-cell populations was shared at the level of the 17 major populations and did not differ by later documented BTI status.

**Figure 5:**
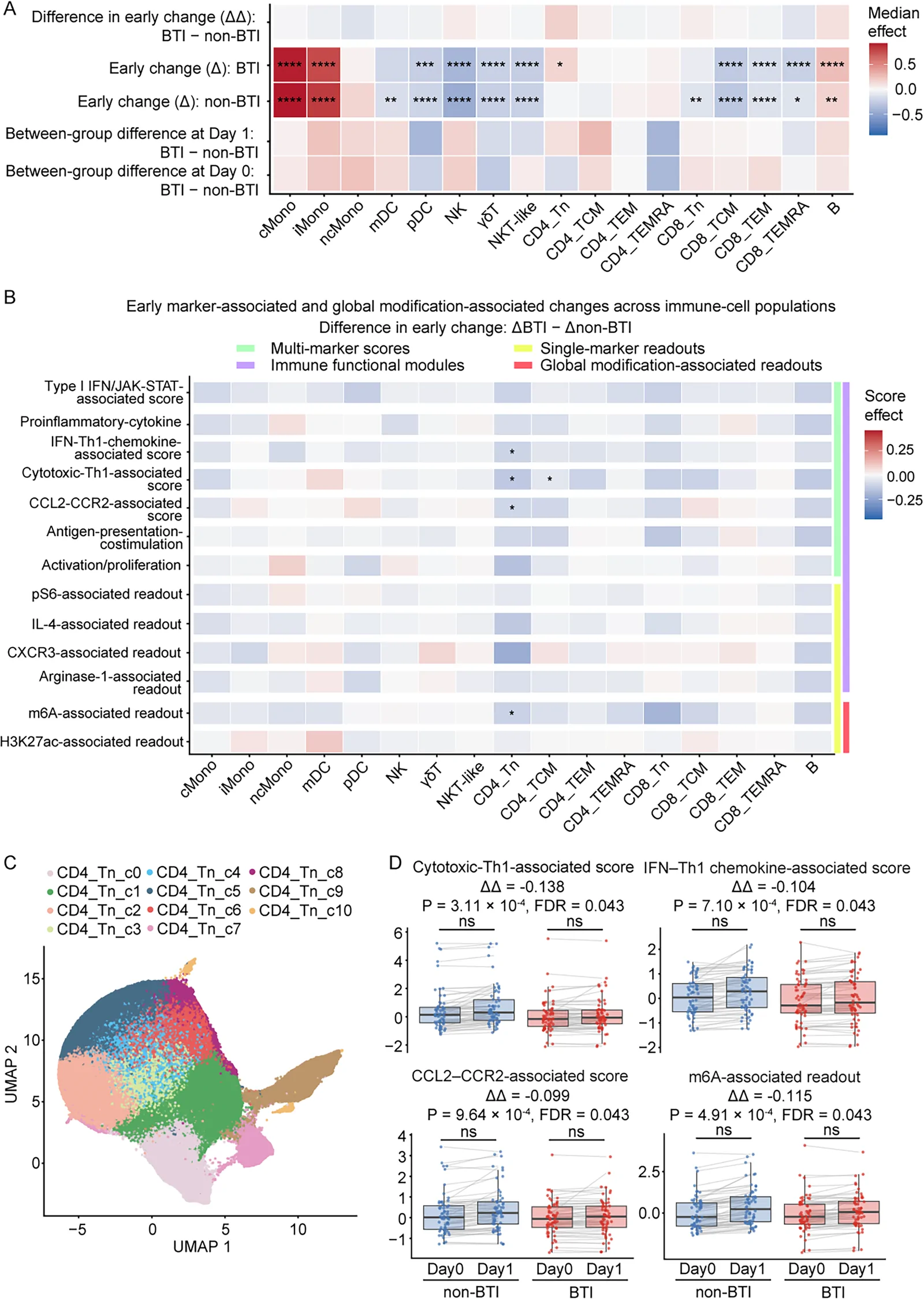
Differential early CD4_Tn marker-associated and m6A signal changes are associated with subsequent BTI. **(A)** Heatmap summarizing major immune-cell abundance differences between BTI and non-BTI participants. Rows show the between-group differences at Day 0 and Day 1, the within-participant early change from Day 0 to Day 1 in the non-BTI and BTI groups, and the between-group difference in early change. Early change was defined as Δ = Day 1 − Day 0, and the difference in early change was defined as ΔΔ = Δ_BTI − Δ_non-BTI. Tile color indicates the direction and magnitude of the displayed median effect in CLR-transformed abundance. Between-group comparisons at Days 0 and 1 and comparisons of Δ values were performed using two-sided Wilcoxon rank-sum tests; within-group Day 0-to-Day 1 changes were evaluated using paired Wilcoxon signed-rank tests. BH-FDR correction was applied within each comparison family. **(B)** Heatmap showing BTI versus non-BTI differences in participant-level early feature changes (Δ) across 17 major immune-cell populations. Thirteen prespecified marker-associated composite/single-marker readouts and global modification-associated readouts were evaluated, yielding 221 feature–cell-population tests (13 × 17). BH-FDR correction was applied across the complete set of 221 tests. Tile color indicates the median between-group difference in Δ (BTI minus non-BTI). Asterisks indicate feature–cell-population combinations with BH-adjusted q< 0.05. **(C)** UMAP visualization of unsupervised clustering within the CD4_Tn compartment, resolving 11 phenotypically distinct subclusters, designated CD4_Tn_c0 through CD4_Tn_c10. **(D)** Participant-level Day 0 and Day 1 distributions for the four CD4_Tn features among the five feature–cell-population combinations that remained significant in the primary 221-test global screen shown in panel **(B)** Lines connect paired samples from the same participant. Panel D provides a focused visualization of the CD4_Tn findings and does not constitute a separate statistical screen. The nominal P values and BH-adjusted q values shown are carried forward from the corresponding BTI-versus-non-BTI comparisons of participant-level Δ values in panel B; no additional multiple-testing correction was performed within these four selected CD4_Tn features. Asterisks in panel A indicate adjusted significance: *FDR< 0.05, **FDR< 0.01, ***FDR< 0.001, and ****FDR< 0.0001. BTI, breakthrough infection; non-BTI, no documented breakthrough infection; CD4_Tn, CD4 naive T cells; CLR, centered log-ratio; Δ, within-participant Day 1-minus-Day 0 change; ΔΔ, difference in early change between BTI and non-BTI groups; m6A, N6-methyladenosine.

In contrast, analysis of marker-associated composite scores and global modification readouts identified a focused set of differences within CD4 T-cell populations (**Figure 5B**). The screen comprised all 13 prespecified feature readouts across all 17 major immune-cell populations (221 tests), with BH-FDR correction applied across the complete set. Five feature–cell-population combinations remained significant after this global correction. Four were within the CD4_Tn compartment—the IFN–Th1 chemokine-associated, cytotoxic–Th1-associated, and CCL2–CCR2-associated marker scores and the m6A-associated readout—and one additional association involved the cytotoxic–Th1-associated score in CD4_TCM cells. Relative to non-BTI participants, individuals who subsequently developed BTI showed smaller (more negative) Day 0-to-Day 1 changes in each of these features. Four of the five FDR-significant combinations were concentrated within CD4_Tn, suggesting that differences in early marker-associated trajectories within this compartment were a prominent feature associated with subsequent BTI.

Because robust standardization can amplify differences in low-intensity channels, we additionally examined the pre-standardization signal distributions of the constituent markers within CD4_Tn cells (**Supplementary Figure S3** and **Supplementary Table S6**). Signal intensity varied substantially among markers. Granzyme B showed a strong non-zero distribution, while MCP-1, CXCL9, and CD57 showed measurable intermediate-level signals. In contrast, IFN-γ, CD86, CXCR3, and, to a lesser extent, CXCL10 showed relatively low absolute signal intensities. These findings indicate that the composite scores were not uniformly driven by near-zero signals, while also supporting more cautious interpretation of modules containing lower-intensity constituent markers.

Unsupervised analysis further demonstrated substantial phenotypic heterogeneity within the CD4_Tn compartment, resolving 11 subclusters (**Figure 5C**). Because the BTI-associated feature differences were identified at the total CD4_Tn level, these associations were retained at the bulk CD4_Tn level rather than assigned to individual subclusters. To provide a focused visualization of the CD4_Tn findings, participant-level Day 0 and Day 1 values for the four CD4_Tn features among the five FDR-significant feature–cell-population combinations are shown in **Figure 5D**, stratified by subsequent BTI status. These four CD4_Tn features—the cytotoxic–Th1-associated, IFN–Th1 chemokine-associated, and CCL2–CCR2-associated marker scores and the m6A-associated readout—remained significant after BH correction across the complete set of 221 feature–cell-population tests in **Figure 5B**. **Figure 5D** was not treated as a separate statistical screen; the nominal P values and BH-adjusted q values shown for the between-group differences in participant-level Δ were carried forward directly from the corresponding **Figure 5B** tests without additional re-testing or re-adjustment. The participant-level distributions illustrate consistently smaller Day 0-to-Day 1 changes in the BTI group across these four globally FDR-significant CD4_Tn features.

As a sensitivity analysis, we additionally modeled participant-level Δ values for the four CD4_Tn features as a function of subsequent BTI status with adjustment for age, sex, and vaccine product using HC3 robust standard errors. The direction of the BTI-associated differences remained consistent for all four features after covariate adjustment (**Supplementary Figure S4A**). Participant-level Δ distributions and corresponding between-group effect estimates are shown in **Supplementary Figure S4B**.

Together, these findings identify early CD4_Tn marker-associated signatures and m6A-associated signal changes as features associated with subsequent BTI status.

## 4 Discussion

In this longitudinal systems-immunology study, we characterized the early peripheral immune response to COVID-19 mRNA booster vaccination and examined its relationship with subsequent humoral immunity and breakthrough infection. Several findings emerged. First, vaccination induced a rapid but heterogeneous reorganization of the circulating immune landscape within 24 h, characterized by increased relative representation of classical and intermediate monocytes and decreased relative representation of pDC, NK, CD8 T-cell, and unconventional T-cell populations. Second, these abundance changes were accompanied by lineage-specific marker-level changes, including changes in inflammatory, migratory, activation-associated, and global H3K27ac and m6A signals. Third, although the abundance of the major CD4 T-cell populations remained comparatively stable, marker-level changes were detectable. CD86 and CXCR3 showed among the largest statistical changes in CD4_Tn cells, although both exhibited relatively low absolute signal intensity in the subsequent per-marker QC analysis and are therefore interpreted cautiously. Fourth, early m6A- and H3K27ac-associated changes correlated with relative-abundance changes of selected immune populations, but their relationships with subsequent neutralizing-antibody responses were limited after correction for multiple testing. Finally, major-cell abundance trajectories did not distinguish participants who subsequently experienced breakthrough infection from those without documented BTI; instead, the most focused outcome-associated differences involved early marker-associated composite scores and the m6A signal within the CD4_Tn compartment. Collectively, these results show that Day 0-to-Day 1 marker changes captured outcome-associated variation that was not evident from major-cell abundance alone.

The rapid cellular changes observed at Day 1 are consistent with previous systems-vaccinology studies demonstrating that mRNA vaccination triggers an early, coordinated innate response before the full development of adaptive immunity. Following BNT162b2 vaccination, early interferon-associated transcriptional programs, inflammatory monocyte responses, and changes in circulating immune-cell populations have been detected within the first few days after vaccination. Systemic increases in IL-15, IFN-γ, and CXCL10 have also been linked to effective vaccine responses, supporting a role for early cytokine and chemokine networks in coordinating subsequent immunity (Bergamaschi et al., 2021; Li et al., 2022). In the present study, the opposing Day 1 trajectories of monocyte, pDC, and NK-cell populations extend these observations by showing that the early response involves not only generalized activation but also substantial shifts in relative abundance among circulating immune compartments.

Classical and intermediate monocytes displayed the largest positive changes in relative abundance, accompanied by changes in MCP-1/CCL2, CCR2, arginase-1, CD38, and H3K27ac-associated signals. The MCP-1 and CCR2 signals are compatible with, but do not demonstrate, CCL2–CCR2-mediated mobilization or recruitment during rapid reorganization of the myeloid compartment following vaccination. In contrast, the decreased relative abundance of circulating pDCs and NK cells may reflect tissue recruitment, vascular margination, changes in survival, or compositional effects arising from the increase in other populations. Thus, these findings should not be interpreted as demonstrating absolute monocyte expansion or depletion of pDCs and NK cells. Because cellular abundance was measured as a proportion of live single cells and analyzed after CLR transformation, the results describe changes in relative composition rather than absolute cell numbers. Because each CLR coordinate is defined relative to the sample-wise geometric mean, the direction of smaller shifts is also reference-dependent. We therefore restrict directional biological interpretation to the most pronounced compositional changes and describe smaller effects conservatively as modest or comparatively stable. Absolute counting, chemokine-receptor trafficking assays, and paired sampling of draining lymph nodes or injection-site tissues would be required to establish the cellular movements underlying these peripheral-blood trajectories.

The early T-cell response displayed a complementary pattern. Changes in circulating abundance were concentrated in CD8 and unconventional T-cell populations, whereas the four major CD4 T-cell subsets were comparatively stable. Previous studies have shown that SARS-CoV-2 mRNA vaccination rapidly mobilizes antigen-specific CD8 T cells and induces transient effector activation followed by contraction and preservation of the memory pool (Oberhardt et al., 2021; Reinscheid et al., 2022). Therefore, the decreased relative representation of several CD8 and unconventional T-cell subsets at Day 1 may reflect early trafficking or redistribution rather than an impaired cellular response. Moreover, the present CyTOF panel measured total phenotypic populations rather than spike-specific cells; changes in the much smaller antigen-specific compartment may occur in parallel with different trajectories in the total population.

Importantly, stable abundance did not imply molecular quiescence. Despite minimal change in total CD4_Tn abundance, CD86 and CXCR3 showed the most prominent marker-level changes in this population. However, because the compartment was defined phenotypically and no antigen-specific assay was applied, these marker changes should not be equated with spike-specific CD4 T-cell activation. The CD86 signal warrants orthogonal confirmation because CD86 is not a canonical marker of conventional T cells. Rapid induction of antigen-specific CD4 T cells, including Th1- and T follicular helper-like responses, has been consistently observed after mRNA vaccination and has been linked to coordinated CD8 T-cell and antibody responses (Painter et al., 2021; Mudd et al., 2022). Vaccine-induced CD4 T-cell responses also retain substantial recognition of SARS-CoV-2 variants, supporting their potential contribution when antibody recognition is reduced by viral evolution (Tarke et al., 2021; Short and Wall, 2021). The CXCR3 signal is compatible with, but does not establish, an early change in migratory or Th1-associated potential.

A notable feature of this study was the simultaneous antibody-based measurement of global m6A and H3K27ac signals across multiple immune-cell populations. H3K27ac distinguishes active from poised regulatory elements and is dynamically remodeled during innate-cell activation (Creyghton et al., 2010; Kaikkonen et al., 2013; Ostuni et al., 2013). Epigenetic reprogramming of monocytes can also produce altered functional responses to subsequent stimulation, as demonstrated in experimental models of trained immunity (Kleinnijenhuis et al., 2012; Saeed et al., 2014; Arts et al., 2018). In parallel, m6A regulates RNA stability and translation and can modulate type I interferon and interferon-stimulated gene responses (Winkler et al., 2019; McFadden et al., 2021). The correlations observed here between m6A or H3K27ac changes and abundance changes in monocytes, pDCs, NK cells, and selected T-cell subsets are biologically plausible but establish only correlations between global modification signals and relative-abundance changes.

Nevertheless, the current measurements should be interpreted as global modification-associated signals, not definitive evidence of epigenomic or epitranscriptomic reprogramming. CyTOF-based measurements provide a global antibody-derived signal for H3K27ac or m6A but do not identify the modified transcripts, genomic loci, enhancers, or downstream genes involved. In addition, the H3K27ac signal was not normalized to total histone abundance and may therefore partly reflect variation in cellular histone or chromatin content. Similarly, because the CyTOF panel did not include an independent proxy for total cellular RNA, variation in the m6A signal may reflect differences in both total RNA abundance and the abundance or composition of m6A-containing RNA species. The whole-cell m6A measurement also does not distinguish mRNA from rRNA, tRNA, or other RNA classes. Accordingly, these readouts should be interpreted as global modification-associated signals rather than normalized modification fractions.

In addition, correlation between a global modification signal and cell abundance does not establish that the modification caused the abundance change. The correlated measurements may instead reflect a shared activation program, altered cell composition within a manually defined population, or differential trafficking of cells with pre-existing molecular states. The 24-h design also does not include a secondary challenge and therefore cannot establish trained immunity. Future studies integrating single-cell RNA sequencing, chromatin-accessibility profiling, locus-specific H3K27ac assays, and transcript-resolved m6A analysis will be necessary to define the regulatory pathways represented by these signals.

The longitudinal antibody data provide further context for interpreting the early cellular response. Both groups showed broad increases in measured neutralization against the tested SARS-CoV-2 variants, consistent with the capacity of mRNA vaccines to induce persistent germinal-center reactions, T follicular helper-cell responses, affinity maturation, and durable immune memory (Turner et al., 2021; Mudd et al., 2022; Kim et al., 2022; Goel et al., 2021). However, early m6A and H3K27ac changes were not broadly associated with the Day 14 antibody response after correction for multiple testing. Early m6A and H3K27ac changes were not robustly associated with either the Day 14 or Day 180 neutralizing-antibody responses after exclusion of post-BTI measurements and correction for multiple testing. Although several nominal cell population–marker–strain associations were observed, none survived FDR correction. These results suggest that the global Day 1 H3K27ac and m6A signals measured in circulating immune cells do not provide strong evidence of a reproducible relationship with subsequent serum neutralizing-antibody magnitude in this cohort. The nominal correlations should therefore be interpreted as exploratory rather than as evidence of specific molecular determinants of later humoral immunity.

The limited number of corrected associations with antibody responses also highlights the compartmental and temporal complexity of humoral immunity. Germinal-center B-cell selection, T follicular helper-cell support, plasma-cell differentiation, and memory-B-cell evolution predominantly occur in secondary lymphoid tissues over weeks to months, whereas the current cellular profiling captured peripheral blood at a single early post-vaccination time point. Persistent germinal-center reactions and continued somatic hypermutation after mRNA vaccination can generate progressively matured antibody and memory-B-cell responses that are not readily predicted from a global Day 1 marker measured in circulating cells (Turner et al., 2021; Kim et al., 2022; Goel et al., 2021). Therefore, the absence of broad Day 1 associations should not be interpreted as evidence that early cellular events are unimportant, but rather that a single peripheral measurement is unlikely to capture all determinants of subsequent humoral immunity.

Neutralizing antibodies remain an important population-level correlate of protection against symptomatic SARS-CoV-2 infection, as demonstrated by vaccine-efficacy trials and quantitative modeling studies (Khoury et al., 2021; Gilbert et al., 2022). Nevertheless, breakthrough infection is not determined by antibody level alone. Studies of vaccinated cohorts have associated lower peri-infection antibody levels with increased breakthrough risk, but infection also depends on exposure intensity, time since vaccination, circulating variant, mucosal immunity, host characteristics, and behavioral factors (Bergwerk et al., 2021). In the present cohort, the neutralizing-antibody trajectories overlapped substantially, with some strain–time-point differences between BTI and non-BTI participants, and neither cross-sectional major-cell abundance nor major-cell abundance change distinguished the two outcomes. Accordingly, the CD4_Tn association was not accompanied by a uniform between-group difference in major-cell abundance or neutralization across all strains and time points.

The most focused BTI-associated finding was smaller Day 0-to-Day 1 changes in the CD4_Tn IFN–Th1 chemokine-associated, cytotoxic–Th1-associated, and CCL2–CCR2-associated marker scores and in the m6A readout. These four CD4_Tn associations, together with an additional cytotoxic–Th1-associated score in CD4_TCM cells, comprised the five feature–cell-population combinations that remained significant after BH correction across the complete 221-test family. Thus, four of the five FDR-significant associations were concentrated within CD4_Tn. Because major-cell abundance changes did not differ between groups, these findings emphasize participant-level marker-associated changes rather than static major-cell composition. Early CD4 T-cell responses have previously been linked to the coordination and durability of downstream humoral and cellular immunity (Painter et al., 2021; Goel et al., 2021), providing biological context but not establishing that the bulk CD4_Tn signals observed here were antigen specific.

Importantly, the constituent-marker QC demonstrated heterogeneous absolute signal intensities within CD4_Tn cells. Granzyme B was robustly detected, and MCP-1, CXCL9, and CD57 showed measurable signals, whereas IFN-γ and several other channels showed substantially lower intensities. Thus, the composite score associations should be interpreted as marker-associated signatures integrating constituents with differing signal strengths, rather than as evidence that CD4_Tn cells directly acquired cytotoxic, chemotactic, or interferon-producing functions. The module scores represent combinations of measured markers rather than direct functional assays. In addition, although unsupervised clustering demonstrated substantial CD4_Tn heterogeneity, the significant module analysis were conducted in the total CD4_Tn population and therefore cannot yet be assigned to a specific CD4_Tn subcluster.

This study has several strengths, including a paired within-participant design, frequency-balanced BTI and non-BTI groups, high-dimensional profiling of multiple innate and adaptive immune populations, longitudinal neutralization measurements against a broad variant panel, and systematic control for multiple testing. Several limitations should also be acknowledged. First, CyTOF measurements were restricted to peripheral blood at Days 0 and 1 and may not reflect events at the injection site, draining lymph nodes, bone marrow, or respiratory mucosa. Second, relative abundance and CLR-transformed data cannot establish absolute cellular expansion, loss, or tissue migration. Third, the analysis did not directly identify vaccine-specific cells, and some early molecular changes may have occurred in non-antigen-specific bystander populations. Fourth, the global m6A and H3K27ac signals lack transcript- and locus-level resolution and were not normalized to total RNA or total histone content, respectively. They therefore cannot distinguish changes in modification abundance from changes in the corresponding bulk molecular content or establish specific epigenomic or epitranscriptomic regulatory mechanisms. Fifth, the outcome-balanced selection of equal numbers of BTI and non-BTI participants was designed for comparative immune profiling and therefore cannot be used to estimate the incidence or absolute risk of BTI in the parent trial. Sixth, illness-triggered surveillance may have missed asymptomatic or unreported infections; accordingly, non-BTI denotes no documented COVID-19 rather than confirmed absence of infection. In addition, breakthrough infection is influenced by factors beyond measured immune features, including exposure intensity, circulating viral variants, behavioral factors, and other host characteristics that were not fully captured in the present analysis and may contribute to residual confounding. Although the BTI and non-BTI subsets were balanced with respect to vaccine product during participant selection, residual formulation-specific effects cannot be completely excluded. The outcome-selected design and absence of external validation also preclude estimation of predictive performance. Finally, the number of tested cell population–marker–strain combinations was large, and none of the exploratory H3K27ac/m6A–antibody correlations survived correction across the complete testing family. These nominal associations should therefore be interpreted cautiously and require independent replication.

In conclusion, mRNA booster vaccination induced rapid, lineage-specific reorganization of the circulating immune system, accompanied by concurrent changes in cellular composition, marker-associated changes, and global H3K27ac and m6A signals. Major-cell abundance remodeling was largely shared between groups defined by subsequent documented BTI status, whereas differences in early CD4_Tn marker-associated composite scores and m6A signals were associated with documented BTI status. These findings suggest that dynamic early immune states may capture variation not evident from major-cell abundance alone when characterizing inter-individual variation in vaccine responses. Further validation using antigen-specific assays, absolute cell counts, multi-omic profiling, and independent prospective cohorts will be required to establish the biological and clinical relevance of these early CD4_Tn associations.

## Data Availability

The original contributions presented in the study are included in the article/**Supplementary Material**. Further inquiries can be directed to the corresponding authors.
