# Supplementary material for: Mass cytometry of early immune remodeling after mRNA booster vaccination reveals exploratory CD4 naive T-cell associations with documented breakthrough infection
Source: Front Cell Infect Microbiol. 2026 Oct 15;16:1966930. doi: 10.3389/fcimb.2026.1966930 (PMC13716633; doi:10.3389/fcimb.2026.1966930)
Supplement: Supplementary file 1 [file Table1.docx]

# Figure S1 Mass cytometry gating strategy

Representative mass cytometry gating strategy for identification of major immune cell populations. Single cells were identified based on DNA content and event length, followed by exclusion of dead cells and annotation of major immune lineages using lineage and differentiation markers.

# Figure S2 Antibody titration and unstained-blank controls for the mass-cytometry staining and barcoding panels

Antibodies were individually titrated during panel development to determine the working concentrations used for the cohort experiments. For each titration series, cells from the same preparation were processed under the same experimental conditions, with an unstained blank control receiving no antibody staining. Red boxes indicate the antibody concentration selected for the final mass-cytometry panel. (A) Titration of surface-staining antibodies. Titration was initiated at 2 μL per test, followed by serial dilutions as indicated. (B) Titration of intracellular-staining antibodies. Cells underwent the same fixation and permeabilization procedure used for intracellular staining, and titration was initiated at 4 μL per test, followed by serial dilutions as indicated. (C) Titration of the six metal-conjugated anti-CD45 antibodies used for the combinatorial barcoding system (106Cd, 110Cd, 111Cd, 113Cd, 114Cd, and 116Cd). Titration was initiated at 2 μL per test, followed by serial dilutions as indicated. These titration experiments and parallel unstained blank controls were used to assess signal-to-background characteristics and guide selection of the final working antibody concentrations.

# Figure S3 Signal-distribution and lineage-contamination quality control of markers analyzed within the FlowJo-defined CD4_Tn compartment

(A) Pre-standardization median arcsinh-transformed intensities of selected functional, activation-associated, and epigenetic markers across the 17 independently FlowJo-defined major immune-cell populations. (B) Marker signal characteristics within the CD4_Tn compartment. Points indicate median arcsinh-transformed intensity, error bars indicate the interquartile range, and annotations show the percentage of cells with zero raw signal for each marker. (C) Spearman correlations between selected functional-marker intensities and lineage- or activation/APC-associated markers within CD4_Tn cells. Correlation coefficients are shown descriptively to assess whether functional-marker signals were strongly associated with lineage-incongruent or activation-associated events; single-cell P values were not used for inferential conclusions. (D) Representative single-cell distributions of Granzyme B, MCP-1, CXCL9, and IFN-γ in CD4_Tn cells compared with biologically expected reference populations. These reference populations were used to contextualize relative signal intensity and were not considered equivalent to FMO, isotype, or channel-matched unstained controls. All marker intensities shown are pre-standardization arcsinh-transformed CyTOF signals.

# Figure S4 Covariate-adjusted sensitivity analysis of early CD4 naive T-cell marker-score and m6A changes associated with subsequent breakthrough infection

(A) Covariate-adjusted estimates of the association between subsequent breakthrough infection (BTI) status and participant-level Day 0-to-Day 1 changes (Δ) in the four CD4_Tn features highlighted in Figure 5D: the IFN–Th1 chemokine-associated score, cytotoxic–Th1-effector, CCL2–CCR2-associated score, and m6A-associated readout. For each feature, Δ was modeled as a function of BTI status with adjustment for age, sex, and vaccine product. Points indicate the adjusted BTI coefficient (BTI minus non-BTI), and horizontal bars indicate HC3-robust 95% confidence intervals. Negative coefficients indicate smaller Day 0-to-Day 1 changes in the BTI group. Benjamini–Hochberg false-discovery-rate (BH-FDR) correction was applied across the four prespecified readouts. (B) Participant-level distributions of Day 0-to-Day 1 changes (Δ = Day 1 − Day 0) for the same four CD4_Tn features, stratified by subsequent BTI status. Each point represents one participant; violin plots show the distribution of Δ values, and boxplots indicate the median and interquartile range. The displayed effect is the median between-group difference, defined as BTI minus non-BTI, with 95% bootstrap confidence intervals. Nominal P values were obtained using two-sided Wilcoxon rank-sum tests. The BH-adjusted q values shown for these four features were carried forward from the corresponding tests in the primary 221-test Figure 5B analysis; no additional multiple-testing correction was performed within the four selected features. For all four features, the Δ values were lower in the BTI group than in the non-BTI group, consistent with the covariate-adjusted sensitivity analysis in panel A.

BTI, breakthrough infection; non-BTI, no documented breakthrough infection; CD4_Tn, CD4 naïve T cells; Δ, within-participant Day 0-to-Day 1 change; CI, confidence interval; BH-FDR, Benjamini–Hochberg false discovery rate.
